# Supplementary material for: Integrated Proteomics and Metabolomics Link Acne to the Action Mechanisms of Cryptotanshinone Intervention
Source: Front Pharmacol. 2021 Sep 1;12:700696. doi: 10.3389/fphar.2021.700696 (PMC8440807; doi:10.3389/fphar.2021.700696)

1-Stearoyl-2-oleoyl-sn-glycerol 3-phosphocholine (SOPC)

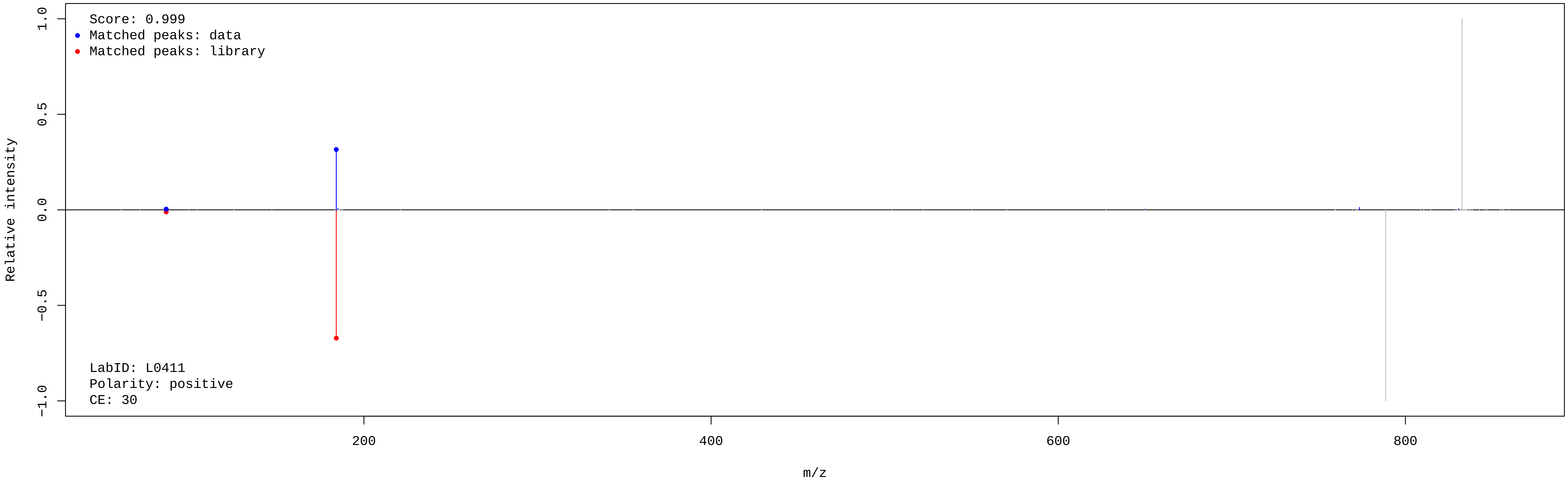

Supplement: Supplementary file 1 [file DataSheet1.ZIP › Supplementary table 1-10 and material 1-3/Material 3-Metlib-MSMS/POS-Metlib-MSMS/Metlib-MSMS/M833T86_forward/0.999,1-Stearoyl-2-oleoyl-sn-glycerol 3-phosphocholine (SOPC),(M-H+2Na)+.pdf]
